# Impact of tuberculosis treatment on health-related quality of life of pulmonary tuberculosis patients: a follow-up study

**DOI:** 10.1186/1477-7525-12-19

**Published:** 2014-02-14

**Authors:** Muhammad Atif, Syed Azhar Syed Sulaiman, Asrul Akmal Shafie, Muhammad Asif, Muhammad Khan Sarfraz, Heng Chin Low, Zaheer-Ud-Din Babar

**Affiliations:** 1 Discipline of Clinical Pharmacy, School of Pharmaceutical Sciences, Universiti Sains Malaysia, Penang, Malaysia; 2 Department of Pharmacy, The Islamia University of Bahawalpur, Bahawalpur, Punjab, Pakistan; 3 Discipline of Social and Administrative Pharmacy, School of Pharmaceutical Sciences, Universiti Sains Malaysia, Penang, Malaysia; 4 Department of Pharmacology, School of Pharmaceutical Sciences, Universiti Sains Malaysia, Penang, Malaysia; 5 Faculty of Pharmacy and Pharmaceutical Sciences, University of Alberta, Edmonton, Canada; 6 School of Mathematical Sciences (Statistical Division), Universiti Sains Malaysia, Penang, Malaysia; 7 School of Pharmacy, University of Auckland, Auckland, New Zealand

**Keywords:** Health-related quality of life, Smear positive pulmonary tuberculosis, SF-36v2 health survey, Longitudinal study, Minimal clinically important difference, Malaysia

## Abstract

**Background:**

At present, much of the attention within tuberculosis (TB) management is spent on microbiological cure, and its impact on health-related quality of life (HRQoL) is either undervalued or seldom considered. The aim of this study was to evaluate the impact of TB treatment on HRQoL of new smear positive pulmonary tuberculosis (PTB) patients. Moreover, we also aimed to determine whether the selected socio-demographic and clinical variables were predictive of variability in the HRQoL scores over time.

**Methods:**

This was a prospective follow-up of new smear positive PTB patients who were diagnosed at the chest clinic of Penang General Hospital between March 2010 and February 2011. All eligible patients (i.e., a new case of smear positive PTB, literate and aged 18 years or above) were asked to self-complete the SF-36v2 questionnaire at the start of their treatment, and then subsequently after the intensive phase and at the end of the treatment. A score on a health domain or component summary measure that was less than 47 norm-based scoring (NBS) point was considered indicative of impaired function within that health domain or dimension. Likewise, an individual having mental component summary (MCS) score ≤ 42 NBS point was considered to be at the risk of depression. Repeated measures ANOVA test was performed to examine how the summary scores varied over time, and to determine whether independent variables were predictive of variability in the physical component summary (PCS) and MCS scores over time.

**Results:**

A total of 216 patients completed the SF-36v2 questionnaire at the start of their treatment. Out of these, 177 and 153 completed the questionnaire at the second and third follow-ups, respectively. The mean PCS scores at the start of the treatment, after the intensive phase and at the end of treatment were 41.9 (SD 5.1), 45.8 (SD 4.8) and 46.0 (SD 6.9), respectively. Similarly, the mean MCS scores at the start of the treatment, after the intensive phase and at the end of the treatment were 39.9 (SD 7.3), 45.0 (SD 6.8) and 46.8 (SD 7.8), respectively. More than 23% of the patients were at the risk of depression at the end of their TB treatment. Patient’s age and being a smoker were predictive of differences in the PCS scores. Similarly, monthly income, being a smoker and TB-related symptoms at the start of the treatment were predictive of differences in the MCS scores.

**Conclusion:**

Although HRQoL improved with the treatment, the scores on component summary measures showed compromised physical and mental health among study patients even at the end of their TB treatment.

## Background

At present, much of the attention within tuberculosis (TB) management is spent on microbiological cure, and its impact on health-related quality of life (HRQoL) is either undervalued or seldom considered [1]. Even, the most recent guidelines on the management of TB are silent on this critical aspect [2]. Existing literature shows that TB has substantial and encompassing impact on HRQoL of infected patients. For example, studies showed that as compared with the general population, TB patients reported deficits in their physical and mental well-being [3-7]. Constitutional TB symptoms especially loss of weight, loss of appetite, fever, fatigue and body pain can adversely affect the patient’s role in a society. With regard to pulmonary tuberculosis (PTB), the patients usually present themselves with a history of chest symptoms such as cough (productive or non-productive), chest pain and hemoptysis. These PTB-specific symptoms can further limit the patient’s role in work and social activities. In some communities, TB patients have to face social rejection and isolation because they are considered to be a source of infection for the healthy individuals [1,8,9]. In a few studies, TB patients themselves reported that they experienced negative emotions such as anxiety and fear [1,8]. Stigmatization and negative emotions resulting from the illness could result in a long-term impairment of patient’s psychosocial well-being [10] which may lead to work absenteeism resulting in loss of productivity and reduced monthly income [11].

The Patients’ Charter for TB Care allows the patients to evaluate the program’s performance [2]. From the patient’s perspective, one of the major performance indicators might be the capability of the National Tuberculosis Program (NTP) to address the physical and mental well-being of the patients. Therefore, the assessment of HRQoL of TB patients as an additional indicator of performance will add value to the NTP.

With regard to the impact of TB treatment on HRQoL, only two longitudinal studies from Asia are available in medical literature [11,12], although none used widely acceptable HRQoL assessment tool. Likewise, published literature shows that, amongst the TB patients in the Malaysian population, there is a paucity of research with regard to HRQoL. With these voids in literature, we conducted a longitudinal study to evaluate the impact of TB treatment on HRQoL of smear positive pulmonary PTB patients. Moreover, we also aimed to determine whether the selected socio-demographic and clinical characteristics were predictive of variability in the HRQoL scores over time.

## Methods

### Study settings

The study was conducted at the chest clinic of Penang General Hospital (PGH), which is the first health care facility in Malaysia since 1961. At present, it is the second largest government hospital with a capacity of 1,107 beds, and acts as a point of reference tertiary health care facility in the Northern Region of Malaysia.

The chest clinic of PGH has three full time chest consultants and a minimum of five to six medical officers. In addition, the chest clinic has dedicated paramedic staff committed to providing quality care to the patients. The chest clinic has well equipped TB diagnostic laboratory where the specimens of TB suspects and existing TB patients are investigated using sputum smear examination, culture, nucleic acid amplification tests and drug sensitivity testing. The radiology and pathology departments of PGH also provide routine investigation services to TB patients.

The confirmed cases of TB are advised to take their TB medication either at the chest clinic or at a primary health care unit. In general, patients are advised to take their daily medication under the direct observation of the staff nurse at the chest clinic or a primary health care unit. However, some patients are allowed weekly packing of the daily dose. Weekly packing of the daily dose is only available at the chest clinic. Patients who continue their daily TB medication at a primary health care unit are advised to visit the chest clinic (every two weeks during the intensive phase and every month during the continuation phase) for routine investigation. Patients who default from their treatment for five consecutive days are traced by a team of staff comprising a TB-coordinator, a staff nurse and an attendant.

### Study design and population

This was a prospective follow-up of new smear positive PTB patient diagnosed at the chest clinic of PGH between March 2010 and February 2011. The patients were followed until they completed their TB treatment (i.e., mid of December 2011).

There are approximately 400 new smear positive PTB patients registered at the chest clinic of PGH every year. This number was used as the total population from which the representative sample was drawn. Using an accepted margin of error of 5% and a 95% confidence interval, the minimum sample size was 197 patients.

### Health related qualify to life assessment questionnaire and its scoring

Despite the abundance of questionnaires now available to measure HRQoL in TB patients, the Short Form-36 (SF-36) health survey remains the most valid option [3,4,6,10,13,14]. Psychometric evaluations of the SF-36v2 health survey provide evidence that it is a reliable and valid tool for detecting differences among groups defined by socio-demographic status and social factors [15].

The SF-36v2 has eight scales that gauge eight domains of HRQoL: physical functioning (PF, 10 items), role-physical (RP, four items), role-emotion (RE, three items), bodily pain (BP, two items), vitality (VT, four items), social functioning (SF, two items), general health (GH, five items) and mental health (MH, five items). Eight health domains are further summarized into two; physical component summary (PCS) and mental component summary (MCS) [16,17]. The PF, RP and BP scales strongly correlate with PCS, while the MH, RE and SF scales strongly correlate with MCS [17,18]. The GH and VT scales moderately correlate with physical and mental components [17]. Studies have demonstrated that compared with eight health domains, the PCS and MCS scores are easier to interpret and simpler to analyse statistically [17].

The translations of the SF-36v2 are controlled and managed by the International Quality of Life Assessment (IQOLA) project researchers. These translations are developed using a standard IQOLA translation methodology. This involves multiple independent forward translations by native speakers, reconciliation of the translations into one form, backward translation of this translation into English to check for conceptual equivalence and small qualitative debriefing tests [19]. For this study, the final translated questionnaires (in Malay, Tamil and Mandarin) were the official translation made available through Quality Metric Incorporated.

The standard norm-based scoring (NBS) of eight health domains and summary components were obtained using the standard scoring algorithms (United States weights). The standard scoring algorithms and the standard component summary measures were used based on the recommendations by the developers [17] and other similar studies from Hong Kong and Malaysia [18,20,21]. Quality Metric’s QM Certified Scoring Software was used for scoring the questionnaires.

### Data collection

During the study period, all eligible patients (i.e., a new case of smear positive PTB, literate and aged ≥ 18 years) were asked to self-complete the SF-36v2 questionnaire (either Malay, Mandarin, Tamil or English version) at the start of the treatment, and subsequently after the intensive phase (IP) (second follow-up) and at the end of the treatment (third follow-up) [3,6,13,14]. The patients’ socio-demographic, clinical and treatment-related data were obtained from their medical records and TB notification forms.

Patients were excluded from the study if they were drug abusers or suffering from a major comorbidity (i.e., human immunodeficiency virus (HIV) co-infection, rheumatic disease, psychiatric disease, or malignancy) [4,6].

When any of the enrolled patients did not visit the chest clinic (for second and third follow-ups) on the scheduled clinic hours, attempts were made to trace the patient at the primary health care unit and/or at a later visit. Enrolled patients, who were unable to participate in the study at the second follow-up (e.g., defaulter, transferred out, or not willing to further participate in the study), were not asked to complete the questionnaire at the end of the treatment.

Trained data collectors (three staff nurses at the chest clinic), in clear and simple language, explained the purpose of the study to all eligible participants, and the patients who agreed to participate signed an informed consent form. Throughout the study period, for the data collection, a comfortable place was allotted in the directly observed therapy (DOT) room of the chest clinic. The patients were assured about the confidentiality of the data provided. They were also told about their right to withdraw from the study at any time. Permission to use the questionnaire was obtained from Quality Metric Incorporated, Lincoln, U.S.

### Statistical analysis

Data were analysed using the PASW (Predictive Analysis SoftWare, version 19.0. Armonk, NY: IBM Corp.). All continuous variables were reported as means (SD). Categorical variables were described using counts and proportions (%). During the treatment, a ≥ 3 NBS point change in the health domain scales and component summary measures represented minimal clinically important difference (MCID) [17,22]. The scores on health domain scales and component summary measures (PCS and MCS), ranging from 47 to 53, were considered equivalent to the general population norms. Similarly, a score on a health domain scale or component summary measure that was less than 47 NBS point was considered indicative of impaired function within that health domain or dimension. Likewise, an individual having MCS score ≤ 42 NBS point was considered to be at the risk of depression [17]. General linear model (GLM) repeated measures ANOVA analysis was used to examine how the component summary scores varied over time, and to determine whether other variables (independent variables) were predictive of variability in the PCS and MCS scores over time. Using the commonly used guidelines, effect size was ranked small (partial eta squared = .01), moderate (partial eta squared = .06) and large (partial eta squared = .14) [23]. The significance of the statistical tests was taken at a p-value of < 0.05.

### Ethical approval

The study was registered at the National Medical Research Register (NMRR), Malaysia. The design and conduct of this study was approved by the National Institute of Health (NIH) and by the Medical Research Ethics Committee (MERC), Ministry of Health, Malaysia (Registration ID: NMRR-10-77-5099; MERC reference: dim. KKM/NIHSEC/08/08/04P10-69).

## Results

The study population included 336 new smear positive PTB patients. Of these, 216 completed the SF-36v2 questionnaire at the start of their treatment. The remaining 126 patients were either judged ineligible (i.e., illiterate, foreigner, drug abusers, and/or HIV positive) or unwilling to participate in the study. Out of 216 patients, 177 and 153 completed the questionnaire at the end of second and third follow-ups, respectively. Consequently, the overall dropout rate was 29.2%. The dropout of the patients during these phases of the treatment were either due to their refusal to participate further in the study, or due to their death, default or transfer to other health care facilities. Table 1 and Table 2 describe the socio-demographic and clinical characteristics of the study patients, respectively.

**Table 1:** Socio-demographic characteristics of the patients

**Characteristics**	**Patients n (%)**
**Sex**	
Male	147 (68.1)
Female	69 (31.9)
**Age ≥ 45 years**	
Yes	126 (58.3)
No	90 (41.7)
**Ethnicity**	
Malay	77 (35.7)
Chinese	118 (54.6)
Indian	21 (9.7)
**Marital status**	
Single	64 (29.6)
Married	134 (62.0)
Widow/divorced	18 (8.3)
**Level of education**	
Primary	104 (48.2)
Secondary	78 (36.1)
University	34 (15.7)
**Smoking status**	
Smoker	108 (50.0)
Non-smoker	108 (50.0)
**Alcoholism**	
Yes	63 (29.2)
No	153 (70.8)
**Employment status**	
Employed	174 (80.6)
Unemployed	42 (19.4)
Monthly income (MYR^*^)	
≤ 1000	76 (35.2)
> 1000	140 (64.8)

^*^Malaysian Ringgit.

**Table 2:** Clinical characteristics of the patients

**Characteristics**	**Patients n (%)**
**Form of TB**	
Smear positive PTB^*^	206 (95.4)
Smear positive PTB^*^ and EPTB^**†**^	10 (4.6)
**History of cough (weeks)**	
< 4	59 (27.3)
≥ 4	157 (72.7)
**Cough type**	
Productive	143 (66.2)
Non-productive	73 (33.8)
**Lung cavities**	
Yes	116 (53.7)
No	100 (46.3)
**Number of TB symptoms at the start of treatment**	
≥ 3	154 (71.3)
< 3	62 (28.7)
**Diabetes**	
Yes	90 (41.7)
No	126 (58.3)
**Hypertension**	
Yes	27 (12.5)
No	189 (87.5)

*Pulmonary Tuberculosis; ^**†**^Extrapulmonary Tuberculosis.

Table 3 describes the NBS of eight health domains at different stages of the treatment. During the treatment, with the exception of the RP scale, a change of ≥ 3 NBS point (representing MICD) was observed for all health domains. At the baseline and at the end of the IP, the mean scores for all health domains (except for the VT at the end of the IP) were less than 47 NBS points. In a similar fashion, at the end of the treatment, the mean scores for all health domains (except for the BP and VT) were less than 47 NBS points.

**Table 3:** SF 36v2 health domain scores at different stages of the treatment using norm-based scoring

Scales^*^	**Mean scores (SD)**		
	**Start of treatment**	**End of intensive phase**	**End of treatment**
PF	41.3 (8.1)	45.5 (7.8)	45.5 (8.7)
RP	40.0 (8.1)	43.9 (7.8)	42.7 (7.6)
BP	41.6 (8.3)	46.3 (8.3)	48.5 (8.5)
GH	39.4 (7.9)	44.3 (7.7)	44.3 (10.0)
VT	43.4 (8.2)	50.0 (8.4)	51.8 (9.2)
SF	40.3 (8.5)	43.1 (9.2)	45.2 (8.6)
RE	37.0 (10.6)	41.4 (9.4)	41.9 (9.2)
MH	40.7 (8.8)	46.6 (8.1)	46.8 (7.6)

*Scales: PF (physical functioning), RP (role-physical), BP (bodily pain), GH (general health), VT (vitality), SF (social functioning), RE (role-emotion), MH (mental health).

Table 4 describes the mean PCS and MCS scores for the study patients. Throughout the treatment, both PCS and MCS scores were less than the lower cut-off point of the general population norms. However, during the treatment, MCID (a change of ≥ 3 NBS point) was observed for both component summary measures. An important finding of this study was the risk of depression in 67.1% patients at the start of their TB treatment. Similarly, 35.0% and 23.5% patients were at the risk of depression after the IP and end of the treatment, respectively.

**Table 4:** Changes in the mean summary scores: minimal clinically important difference estimates

Summary measures^*^	**Mean scores (SD)**			**Mean change in scores**^**†**^
	**Start of treatment**	**End of intensive phase**	**End of treatment**	
PCS	41.9 (5.1)	45.8 (4.8)	46.0 (6.9)	4.1
MCS	39.9 (7.3)	45.0 (6.8)	46.8 (7.8)	6.9

*Summary measures: PCS (physical component summary), MCS (mental component summary); ^†^Mean change in summary scores from start to end of tuberculosis treatment.

GLM repeated measures ANOVA test was performed to compare the component summary scores at the start of the treatment, at the end of the IP and at the end of the treatment. Table 5 presents the mean scores for the component summary measures. The analysis revealed a significant effect for PCS and MCS.

**Table 5:** Changes in the mean summary scores: repeated measures ANOVA analysis

Summary measures^*^	**Stages**	**N**	**Mean scores (SD)**
	Start of treatment	153	42.3 (4.8)
PCS^†^	End of intensive phase	153	45.7 (4.3)
	End of treatment	153	46.0 (6.9)
	Start of treatment	153	39.8 (7.4)
MCS^‡^	End of intensive phase	153	44.6 (6.7)
	End of treatment	153	46.8 (7.8)

*Summary measures: PCS (physical component summary), MCS (mental component summary); ^†^Wilk’s Lambda = .62, *F* (2, 151) = 46.33, p < 0.0005, multivariate partial eta squared = .38; ^‡^Wilk’s Lambda = .60, *F* (2, 151) = 49.51, p < 0.0005, multivariate partial eta squared = .40.

Table 6 describes that patient’s age (F = 3.229, df = 1.731, 223.281) interacted with time to predict trends in the PCS scores. Similarly, monthly income (F = 3.710, df = 1.726, 222.640) and hypertension (F = 4.430, df = 1.726, 222.640) interacted with time to predict trends in the MCS scores.

**Table 6:** Test of within-subjects effects for the summary scores: general linear model repeated measures ANOVA analysis

**Source**	**df**	**F**	**p-value**	**Partial eta squared**
Measure: physical component summary^*^				
Time* Age ≥ 45 years	1.731	3.229	0.049	.024
Time* Unemployment	1.731	.106	0.873	.001
Time* Monthly income > 1000 MYR	1.731	2.163	0.125	.016
Time* Diabetes	1.731	.108	0.871	.001
Time* Hypertension	1.731	1.225	0.292	.009
Time* Smoker	1.731	.481	0.592	.004
Time* Productive cough	1.731	1.189	0.302	.009
Time* ≥ 3 TB symptoms at the start of the treatment	1.731	1.930	0.154	.015
Error	223.281			
Measure: mental component summary^*^				
Time* Age ≥ 45 years	1.726	.377	0.655	.003
Time* Unemployment	1.726	.615	0.519	.005
Time* Monthly income > 1000 MYR	1.726	3.710	0.032	.028
Time* Diabetes	1.726	.630	0.511	.005
Time* Hypertension	1.726	4.430	0.017	.033
Time* Smoker	1.726	1.106	0.326	.009
Time* Productive cough	1.726	.759	0.452	.006
Time* ≥ 3 TB symptoms at the start of the treatment	1.726	1.804	0.172	.014
Error	222.640			

*Greenhouse-Geisser values as sphericity cannot be assumed (p < 0.0005).

**Figure 1:**
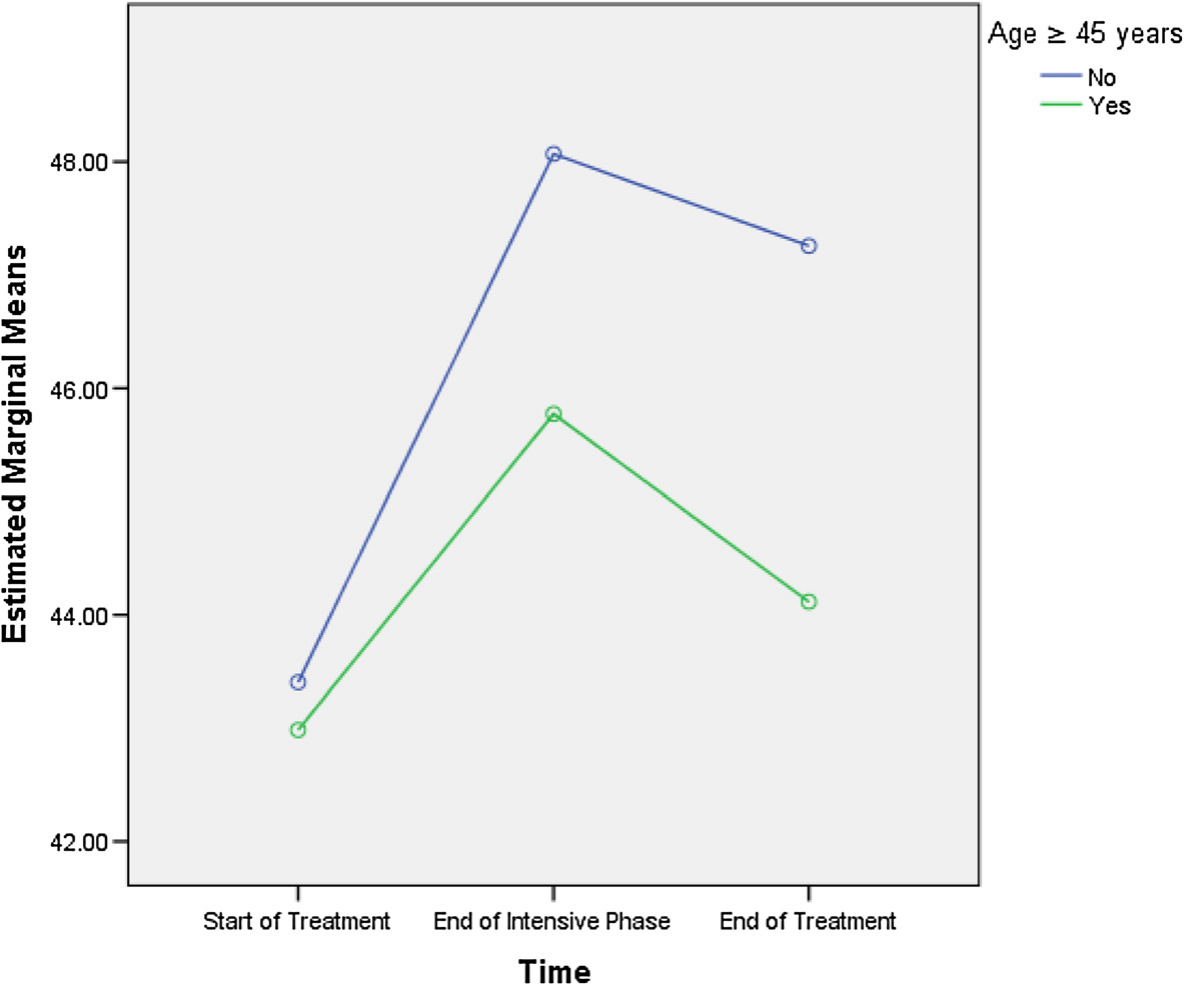
Age more than or equal to 45 years: differences in estimated marginal means of physical component summary.

**Figure 2:**
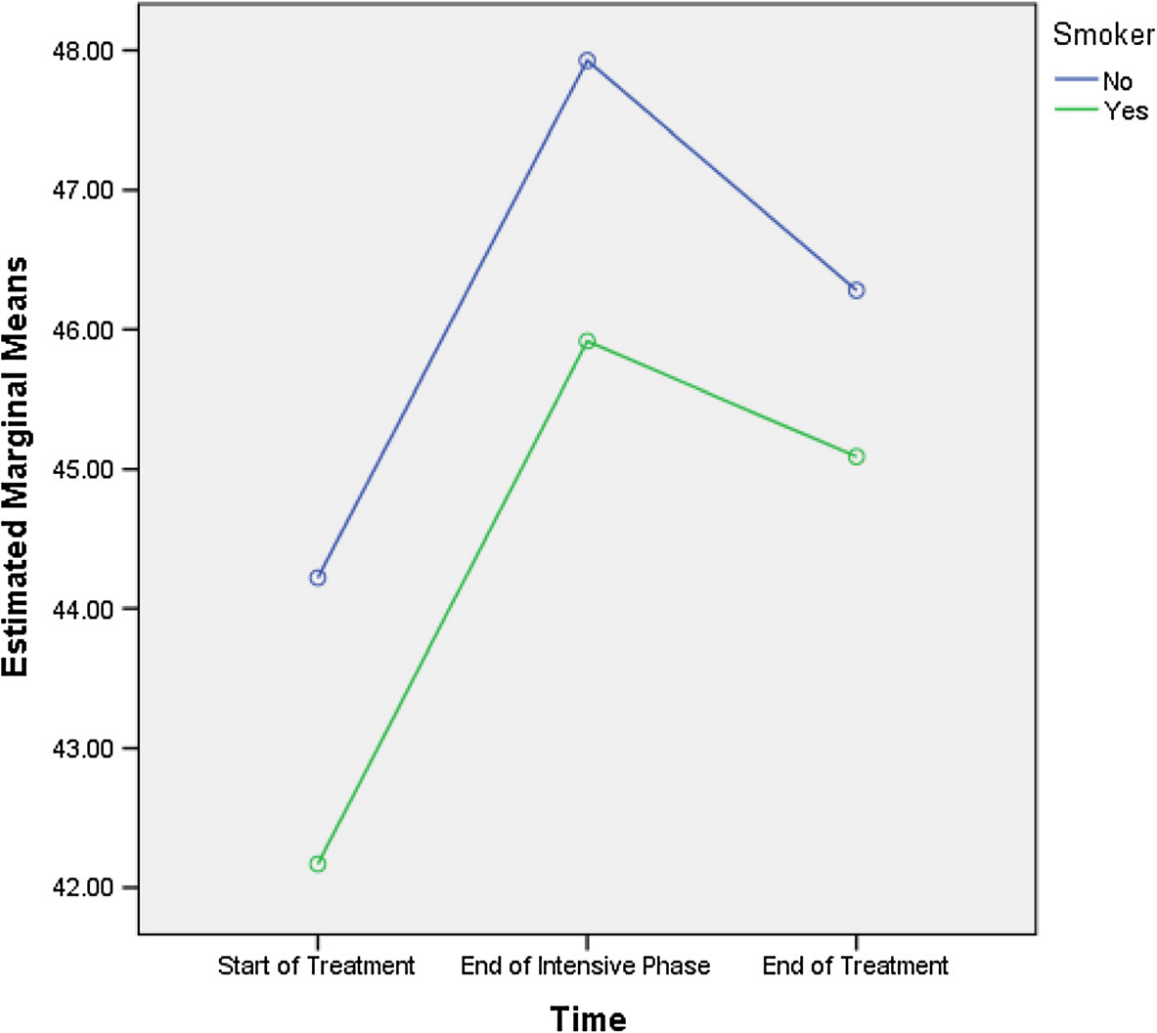
Being a smoker: differences in estimated marginal means of physical component summary.

Table 7 shows that age ≥ 45 years (F = 9.805, df = 1, 129) and being a smoker (F = 9.664, df = 1, 129) were predictive of differences in the PCS scores. As the differences between the groups at time point 2 and 3 were largely reflective of the differences that existed at baseline (time 1), it seems that the differences (in the PCS scores) that existed between groups were more likely to be attributed to the differences in composition of the groups (Figures 1 and 2).

**Table 7:** Test of between-subjects effects for the summary scores: general linear model repeated measures ANOVA analysis

**Source**	**df**	**F**	**p-value**	**Partial eta squared**
**Measure: physical component summary**				
Age ≥ 45 years	1	9.805	0.002	.071
Unemployment	1	.252	0.617	.002
Monthly income > 1000 MYR	1	3.019	0.085	.023
Diabetes	1	1.767	0.186	.014
Hypertension	1	.003	0.956	.000
Smoker	1	9.664	0.002	.070
Productive cough	1	3.271	0.073	.025
≥ 3 TB symptoms at the start of the treatment	1	2.799	0.097	.021
Error	129			
**Measure: mental component summary**				
Age ≥ 45 years	1	.002	0.968	.000
Unemployment	1	.058	0.810	.000
Monthly income > 1000 MYR	1	10.817	0.001	.077
Diabetes	1	.057	0.811	.000
Hypertension	1	1.726	0.191	.013
Smoker	1	7.078	0.009	.052
Productive cough	1	3.748	0.055	.028
≥ 3 TB symptoms at the start of the treatment	1	7.273	0.008	.053
Error	129			

**Figure 3:**
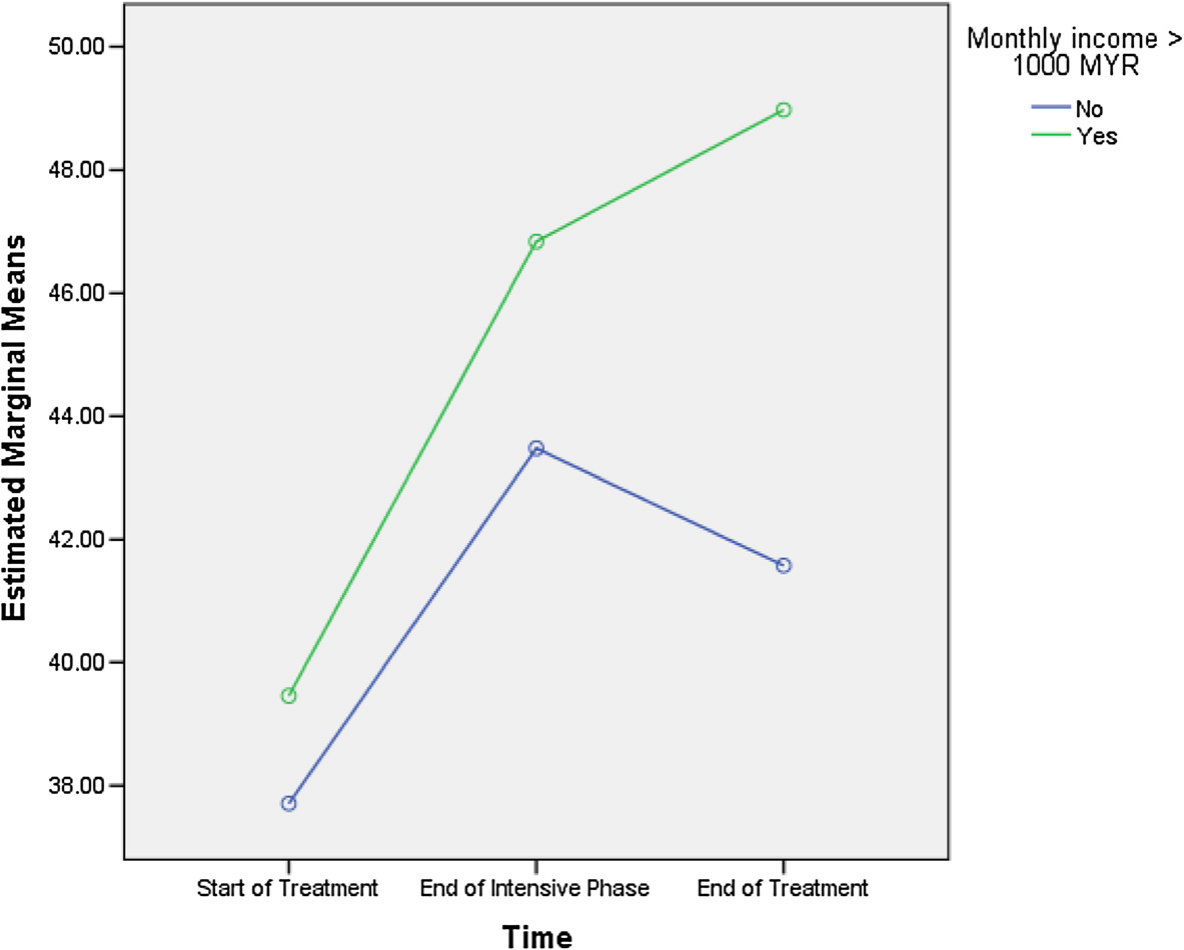
Monthly income more than 1000 MYR: differences in estimated marginal means of mental component summary.

**Figure 4:**
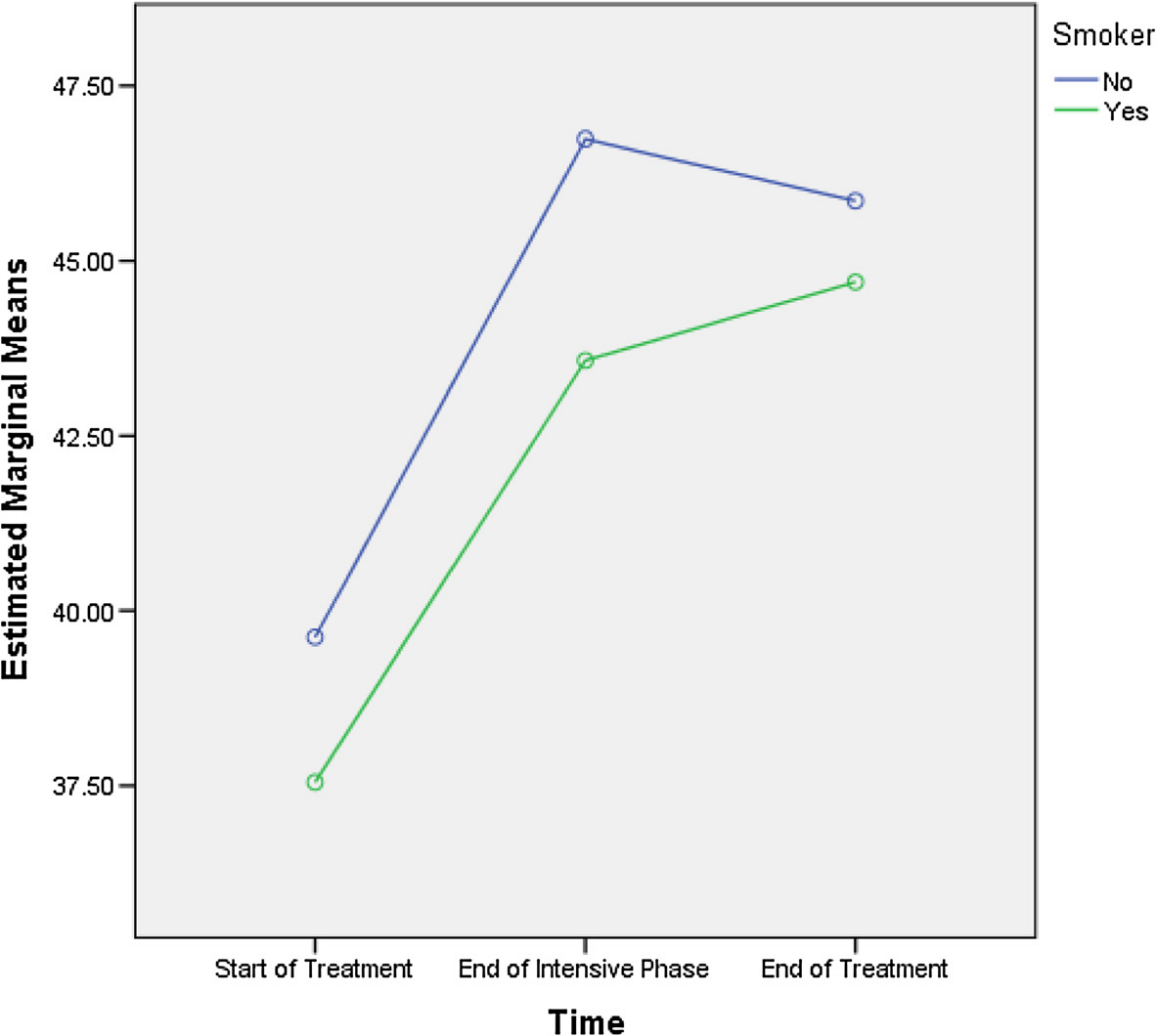
Being a smoker: differences in estimated marginal means of mental component summary.

**Figure 5:**
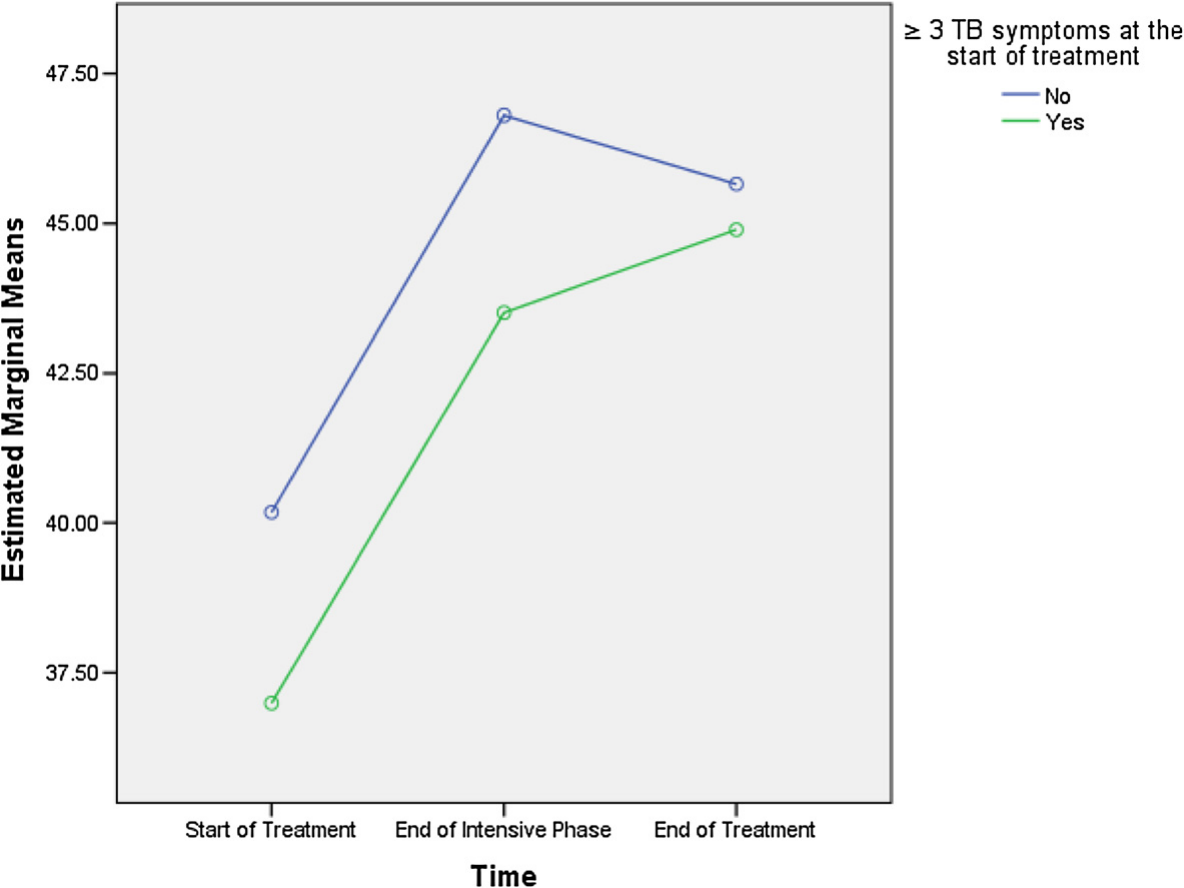
Three or more tuberculosis symptoms at the start of the treatment: differences in estimated marginal means of mental component summary.

Monthly income more than 1000 MYR (F = 10.817, df = 1, 129), being a smoker (F = 7.078, df = 1, 129) and ≥ 3 TB-related symptoms at the start of the treatment (F = 7.273, df = 1, 129) were predictive of differences in the MCS scores (Table 7). As the differences between the groups at time point 2 and 3 were largely reflective of the differences that existed at baseline (time 1), it seems that the differences (in the MCS scores) that existed between groups were more likely to be attributed to the differences in composition of the groups (Figures 3, 4 and 5).

## Discussion

Only a few studies in the literature prospectively studied TB patients’ HRQoL during the whole treatment period [6,11,12,24]. Out of these, only two studies were conducted in Asia [11,12]: one used a DR-12 questionnaire and the other used a modified version of the SF-36 health survey to quantify HRQoL in TB patients. These findings highlighted the existence of a gap in literature with regard to a follow-up study using a validated tool to gauge the impact of TB treatment on HRQoL of smear positive PTB patients.

In the present study, 216 smear positive TB patients completed the SF-36v2 questionnaire at the start of the treatment. Out of these, 153 respondents completed the questionnaire at the end of the treatment. The study’s sample size was larger than all above cited follow-up studies, with the exception of one study which was conducted in India [11]. One of the major reasons for the higher enrollment rate in above cited Indian study might be attributed to the higher incidence rate of TB in India.

The findings of our study showed that there were clinically and statistically significant improvement in the scores on health domain scales (except for the RP) and summary measures. However, despite these improvements following TB treatment, the mean PCS and MCS scores, at the end of the treatment, were less than 47 NBS points. This indicated compromised health. Similarly, at the end of the treatment, scores on all health domain scales (except for the VT and BP) were less than 47 NBS points. These findings clearly suggested that although TB treatment had significantly improved the patients’ perception about their health, altered HRQoL continued even after the completion of the treatment. Similar to current study’s findings, Marra and associates reported that compared with the U.S. general population norms, patients with active TB had worse scores on the SF-36 scales at the end of the treatment [6]. A few other studies also reported almost similar findings [3,10].

With regard to HRQoL at the start of the treatment, the scores on health domain scales and summary measures were less than 47 NBS points. The RE and GH scales were the most affected health domains which meant that the patients had severe problems in performing their daily life activities due to emotional stress. Similarly, it also suggested that the patients rated their overall health as poor, and even expected it to get worse [17]. At the start of the treatment, compared with PCS, lower MCS scores showed that the patients experienced more psychological distress and role limitation due to emotional problems than the physical problems [17]. In line with the current study’s findings, a study from Canada also reported lower HRQoL scores on SF-36 health domain scales and component summary measures. The Canadian study also reported that, at the start of the treatment, the RE and RP were among the most affected health domains [6]. Likewise, a study from the United Kingdom (UK) reported compromised HRQoL across SF-36 health domain scales and component summary measures [13]. However, contrary to the findings of our study, earlier studies [6,13] showed that, at the start of the treatment, physical health was more affected than the mental health.

With regard to HRQoL scores at the end of the IP, the findings of this study showed that, except for the SF, a change of ≥ 3 NBS point was observed for all health domain scales and summary measures. These findings showed that, although HRQoL scores were less than the general population norms (except for the VT); TB treatment had significantly improved HRQoL of the patients. A similar trend in the improvement of HRQoL scores in association with TB treatment was reported elsewhere [13,24]. Contrary to this, a study from China showed that, at the end of the IP, the mean RE, BP and SF scores were not different from those of the controls [3]. The differences in patients’ HRQoL levels among these studies might be either due to inconsistency in patients’ characteristics, differences in cultures and mode of data collection related to HRQoL, or attributable to variation in the NTP’s performance. With respect to patients’ HRQoL at the end of TB treatment, the findings of the current study were in accordance with most of the earlier studies in the same area [3,6,14,24].

Our study findings also showed that, at the start of the treatment, more than 67% patients were at the risk of depression (MCS score ≤ 42 NBS point). Although, proportion of the patients with the risk of depression decreased with the TB treatment, 23.5% patients were still at the risk of depression after the completion of their treatment. Similar to this study’s findings, a UK study reported high level of depression and anxiety among the patients at the time when they were diagnosed with TB. However, depression levels among the patients returned to normal during TB treatment. This was possibly because the clinicians in the UK were aware of non-medical aspects of the disease [13]. However, the risk of depression in approximately 24% patients indicated that the clinicians and NTP managers in current clinical setting had underappreciated the TB’s impact on the mental health of TB patients. In a similar fashion, qualitative studies from Canada and the U.S. showed that, owing to curable nature of the disease, the clinicians underestimated the impact which TB had on the patients’ HRQoL [1,8].

In this study, patient’s age and being a smoker were predictive of differences in the overall PCS scores (Table 7). Figure 1 demonstrates that compared with the patients aged less than 45 years, the mean PCS scores for the patients who were ≥ 45 years of age were lowest at every time point. Similarly, Figure 2 demonstrates that compared with the smokers, the mean PCS scores for the non-smokers were highest at every time point. These findings demonstrated that the differences between the groups at time point 2 and 3 were largely reflective of the differences that existed at baseline (time 1), rather than the varying impact of TB treatment. A higher probability of lower PCS scores in elderly patients might be associated with their old age and the expected decline in physical health with increasing age [17,25,26]. Similar to the findings of our study, a Chinese study reported comparable association between age and HRQoL scores [3]. Along the same lines, a study from Canada also reported lower PCS scores in elderly TB patients [6]. Contrary to our findings, an Indian study showed that literacy, being employed and absence of TB symptoms at the end of the treatment were associated with higher PCS scores in TB patients [14].

With respect to mental health, monthly income, being a smoker and number of TB-related symptoms at the start of TB treatment were predictive of the overall MCS scores. Figure 3 demonstrates that compared with the patients having monthly income more than 1000 MYR, the mean MCS scores for those earning ≤ 1000 MYR per month were lowest at every time point. Similarly, the non-smokers and the patients with less than three TB-related symptoms at the start of treatment had higher MCS scores at every time point compared with the smokers and those exhibiting ≥ 3 TB-related symptoms, respectively (Figures 4 and 5). These findings were similar to the findings of studies from Canada and India [6,14]. However, a study from China did not find any association between smoking and MCS scores [3].

Smoking is known to adversely affect the immune system, and can render the smokers more susceptible to infections. Similarly, smokers tend to have higher bacillary loads because of compromised immune system. A higher bacillary load can augment the severity of disease which may adversely affect the patients’ perception about their mental and physical health [27].

It is a known fact that people with higher monthly income face low economic hardships. Consequently, compared with the persons falling in the low socio-economic category, they are expected to have better mental satisfaction [28]. In the present study, better MCS scores among the patients with higher monthly income added weight to this argument. Higher MCS scores among patients with higher income were also reported in other studies [25,26].

### Limitations

The findings of our study need to be interpreted in the light of certain limitations:

i. The study only included smear positive PTB patients. Therefore, the study’s results could not be generalized for all types of TB patients. However, from the public health perspective, smear positive PTB patients are the most important group of TB patients and these are the most common group of patients used to evaluate the NTP’s performance.

ii. With regard to HRQoL assessment, patients’ dropouts were observed at the second and third follow-ups. Indeed, this is the limitation inherent in the longitudinal study design. To reduce this study bias, attempts were made to contact the patients at the primary health care units and/or at a later visit, if patients under study were not traceable on the scheduled clinic hours (for second and third follow-ups). Of course one might also think that the patients who dropped out at the second and third follow-ups might have a worse clinical condition which could affect the study’s findings. Nevertheless, medical records of the non-responders (except six patients at the second follow-up) did not show any evidence that might have limited their further participation in this study due to worse clinical condition.

iii. As we used the self-administered version of SF-36v2 health survey, illiterate smear positive PTB patients were unable to participate in the study. Therefore, our findings should be applied with caution to illiterate PTB patients.

## Conclusion

The study’s findings showed that, after the treatment, there were clinically and statistically significant improvements of HRQoL (as measured by SF-36v2) for the TB patients. However, the mean PCS and MCS scores at the end of the treatment were still less than 47 NBS points.

Diminished HRQoL scores among the patients, even at their treatment completion, call upon the urgent attention of NTP managers to collect the patient-reported outcomes data at various stages of the treatment. This strategy will enable the clinicians to take timely actions for addressing physical and/or mental well-being of the patients.

The clinicians should also pay special attention to the identified risk factors for poor HRQoL in TB patients. Doubtless, this could bring about comprehensive client-oriented improvements in the NTP.

## Competing interests

The authors declare that they have no competing interests.

## Authors’ contributions

MAT has a significant contribution in the conception and design of study, data acquisition, data analysis and interpretation, and writing of the manuscript. SASS and AAS have a substantial contribution in the conception and design of the study. AAS, SASS, MKS, ZB revised intellectual content of the manuscript. AAS, MAS contributed in data acquisition and manuscript drafting. HCL, ZB and MKS significantly contributed in statistical analysis and manuscript drafting. Final version of the manuscript was approved by all authors. MAT acts as the guarantor for the overall content. All authors read and approved the final manuscript.
